# Body Mass Index-Related Mortality in Patients with Type 2 Diabetes and Heterogeneity in Obesity Paradox Studies: A Dose-Response Meta-Analysis

**DOI:** 10.1371/journal.pone.0168247

**Published:** 2017-01-03

**Authors:** Yeongkeun Kwon, Hyun Jung Kim, Sungsoo Park, Yong-Gyu Park, Kyung-Hwan Cho

**Affiliations:** 1 Department of Family Medicine, Korea University College of Medicine, Seoul, South Korea; 2 Center for Obesity and Metabolic Diseases, Korea University Anam Hospital, Seoul, South Korea; 3 Institute for Evidence-based Medicine, The Korean Branch of Australasian Cochrane Center, Department of Preventive Medicine, Korea University College of Medicine, Seoul, South Korea; 4 Division of Upper Gastrointestinal Surgery, Department of Surgery, Korea University College of Medicine, Seoul, South Korea; 5 Department of Biostatistics, The Catholic University of Korea College of Medicine, Seoul, South Korea; Medical University Innsbruck, AUSTRIA

## Abstract

**Objective:**

We conducted a systematic review and meta-analysis of studies to quantify the association between body mass index (BMI) and the risks of all-cause and cardiovascular mortality in patients with type 2 diabetes.

**Methods:**

We included studies assessing the impact of BMI on all-cause and cardiovascular mortality in patients with type 2 diabetes. Data were combined using a random-effects dose-response model.

**Results:**

Sixteen cohort studies on all-cause mortality (n = 445,125) and two studies on cardiovascular mortality (n = 92,841) were evaluated in the meta-analysis. A non-linear association was observed between BMI and all-cause mortality among patients with type 2 diabetes. With a BMI nadir of 28–30 kg/m^2^, the risk of all-cause mortality displayed a U-shaped increase. With a BMI nadir of 29–31 kg/m^2^, the risk of cardiovascular mortality exhibited a gradual non-linear increase for BMI > 31 kg/m^2^. Subgroup analyses suggested that study location, diabetes duration, and smoking history may have contributed to heterogeneity among the studies.

**Conclusions:**

An obesity paradox exists for patients with type 2 diabetes with respect to all-cause and cardiovascular mortality. Study location, diabetes duration, and smoking history might contribute to heterogeneity among obesity paradox studies of patients with type 2 diabetes.

## Introduction

Overweight and obesity are associated with impaired glucose metabolism and type 2 diabetes. Weight control is therefore recommended in patients with type 2 diabetes [1]. Recently, however, the survival benefits of overweight or obesity have been reported in patients with type 2 diabetes, as observed in patients with other chronic conditions [2,3]. Understanding the survival benefits of overweight or obesity in patients with type 2 diabetes, i.e., the obesity paradox, is vital for providing lifestyle advice and weight recommendations for these patients.

Although type 2 diabetes is usually associated with overweight and obesity, its prevalence among normal-weight individuals has increased during the past decade to more than 10% of individuals with diabetes in the United States [2,4]. Type 2 diabetes in normal-weight individuals might represent the metabolically obese normal weight phenotype [5]. Some population groups that are more predisposed to normal-weight diabetes exist, such as certain ethnic groups and older populations. Addressing the question of the obesity paradox in patients with type 2 diabetes could provide insight into the genetic and phenotypic mechanisms that contribute to better health outcomes independent of weight status. An understanding of these mechanisms could be useful in weight control intervention.

It has been difficult to compare relevant studies because of the variable body mass index (BMI) cutoff values used to define each BMI level category. A systematic review is required to summarize the available data, and particular attention should be devoted to methodological challenges such as varying BMI level categorizations across studies, variations in the populations, and other important aspects of study characteristics. We therefore conducted a meta-analysis of published epidemiological studies of various populations to quantify the association between BMI and the risks of all-cause and cardiovascular mortality among patients with type 2 diabetes. We also examined the dose-response effect of BMI.

## Materials and Methods

### Data sources and searches

We performed a systematic review of the published scientific literature. Relevant studies published between January 1, 1950 and January 31, 2016, were selected by searching MEDLINE, EMBASE, and the Cochrane Central Register of Controlled Trials. The combined text and Medical Subject Heading search terms were body mass index (e.g., obesity, adiposity, body mass index, body size, overweight, fat mass, body fat, body composition, body weight, BMI, body mass), mortality (e.g., mortality, death, survival, prognosis, paradox, all-cause, cardiovascular disease, ischemic heart disease, coronary heart disease, cerebrovascular disease, cerebrovascular accident, stroke), and diabetes (e.g., diabetes, diabetes mellitus, type 2 diabetes, non-insulin–dependent diabetes mellitus) (S1 Appendix). All potentially eligible studies were considered for review regardless of the primary outcome. A manual search using references from the key articles was performed.

### Study selection

We considered studies for inclusion using the following criteria: (1) all participants had type 2 diabetes as determined by self-reported measurements or clinical diagnosis; (2) the exposure of interest was BMI; (3) the outcome was all-cause or cardiovascular mortality; (4) studies reported adjusted hazard ratios (HRs) or odds ratios with corresponding 95% confidence intervals (CIs); and (5) the study was a prospective or retrospective cohort study. The exclusion criteria were as follows: (1) further publication of any of the included studies; (2) data published only in abstract form; (3) case reports, review articles, and commentary articles; and (4) studies with pediatric participants or pregnant populations.

Two independent investigators (Y.K. and H.J.K.) reviewed the study titles and abstracts. Studies that satisfied the inclusion criteria were retrieved for full-text assessment. There was an agreement value (κ) of 94% for the studies selected by these two investigators for detailed analysis. Disagreements were resolved by a third investigator (K-H.C).

### Data extraction and quality assessment

Extracted data included the first author’s name, study location, year of publication, number of participants, study design, participants’ age and sex, follow-up duration, underweight exclusion, BMI category, numbers of patients and cases in each BMI category, covariates controlled in multivariable analysis, method for assessing height and weight, diabetes duration, and adjusted HRs and corresponding 95% CIs. Adjusted risk estimates that reflected the most comprehensive control were extracted to avoid potential confounding variables. The risk of bias was assessed according to the Newcastle-Ottawa Scale for cohort studies [6]. The study quality assessment addressed the selection adequacy, comparability, and outcomes. The present study was reported in accordance with The Meta-analysis Of Observational Studies in Epidemiology guidelines [7]. Details of the protocol for this systematic review were registered on PROSPERO (International prospective register of systematic reviews), and they can be accessed at www.crd.york.ac.uk/PROSPERO/display_record.asp?ID=CRD42016032931.

### Data synthesis and analysis

We used a random-effects model with a generalized least squares estimation [8] to calculate the summarized risk estimates and 95% CIs for an increase in BMI of five units. When the lowest group was not the referent, HRs and CIs were calculated as relating to the referent for which data were required [9]. To generate an estimate for both sexes combined, the sex-specific estimates were combined using a fixed-effects model. The method described by Greenland and Longnecker [8] was used for the dose-response analysis, and study-specific slopes (linear trends) and 95% CIs were computed from the natural logs of the HRs and CIs across categories of BMI. This method requires the distribution of cases and person-years and the median level of BMI in each category to the corresponding HR for each study. HRs with estimates for at least three quantitative exposure categories are required for this method. The midpoint of each BMI category was utilized if the mean or median BMI for the category was not provided in the study. For studies with an open-ended highest or lowest BMI category, we supposed that the amplitude was the same as the closest adjacent category.

We performed a two-stage, random-effects, dose-response meta-analysis to examine the nonlinear dose-response relationship between BMI and mortality (all-cause and cardiovascular) in patients with type 2 diabetes. Non-linear dose-response curves were plotted using restricted cubic splines for each study with knots fixed at the 10^th^, 50^th^, and 90^th^ percentiles through the distribution [10,11]. Additionally, a generalized least-squares method and a multivariate maximum likelihood method were used to estimate the summary nonlinear dose-response relationship while considering random effects [12]. A *P-*value for nonlinearity was calculated by testing the null hypothesis that the coefficient of the second spline was equal to 0.

Heterogeneity was tested using the Cochrane Q test and quantified using the *I*^*2*^ statistic [13]. For the Q statistic [13], heterogeneity was considered present if *P* < 0.1. Low, moderate, and high heterogeneity were defined as *I*^*2*^ values of 25, 50, and 75%, respectively. To detect publication bias, Egger’s regression test was applied. To investigate the sources of heterogeneity among the included studies, subgroup meta-analyses were conducted according to study location (Western or Asian), year of publication (before or after 2010), number of participants (<10,000 or ≥10,000), study design (retrospective or prospective), age (<65 years or ≥65 years), sex (male or female), years of follow-up (<10 or ≥10), measured or self-reported assessment of weight and height, diabetes duration (incident diabetes: yes or no), exclusion of early death during follow-up period (yes or no), and smoking history (yes or no). Sensitivity analysis was conducted by excluding one study at a time to explore whether the results were driven by one large study or by a study with an extreme result.

Statistical analyses were performed using Stata 13 software (Stata Corp., College Station, TX, USA). Statistical significance was indicated by a two-sided *P* < 0.05.

## Results

### Studies included in the meta-analysis

**Fig 1:**
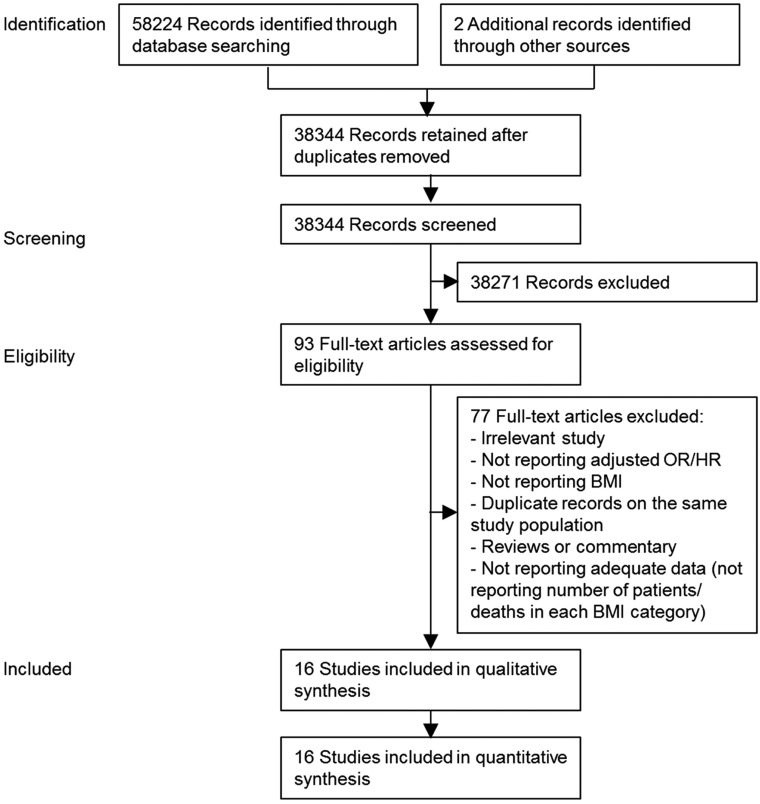
Flow diagram for the selection of studies.

We identified 38,344 studies through electronic searches (Fig 1). Of these, 22,321 were excluded on the basis of titles and abstracts; thus, 93 studies were selected for further assessment. Sixteen studies fulfilled the inclusion criteria, resulting in data for 445,125 participants [3,14–28]. Table 1 lists the summary and study-specific characteristics. Of the included studies, eight, four, and two were conducted in Europe, the United States, and East Asia, respectively, and one study [15] included participants from Europe, East Asia, and the United States. Studies were published as early as 1991. Thirteen studies had a prospective cohort study design, whereas three studies had a retrospective cohort study design. The follow-up duration varied from 3 to 16 years. Five studies [3,14,21,22,24] included patients with incident type 2 diabetes. BMI was measured by medical staff in all but three studies [21,23,27], in which BMI was self-reported by the study participants. S1 Table shows the risk of bias in the included studies.

**Table 1:** Characteristics of Studies Included in the Analysis.

Study(populationor location)	Year of publication(no. of participants)	Design	Baseline age (years), mean	Baseline proportion of women, %	Start of follow-up, year (years of follow-up)	Underweight excluded	Outcome	BMI with best outcome(all-cause mortality,kg/m^2^)	BMI categories(kg/m^2^)	Adjustmentfor covariates	Assessment of weight and height	Diabetesduration(years),mean
Ford et al.(United States)	1991(602)	Prospective	NR	63	1971(10)	No	All-cause mortality	None	<27.8,27.8–31.1,≥31.1	Age, sex, race	Measured	NR
Chaturvedi et al.(Europeans, East Asians, and Native Americans)	1995(2960)	Prospective	47	52	1975(13)	No	All-cause mortality	<26	European<26,26–29,≥29American<29,29–34,≥34Asian<22,22–25,≥25	Age, duration, systolic BP, cholesterol, smoking, retinopathy, and insulin therapy	Measured	NR
Zoppini et al.(Italy)	2003(3398)	Retrospective	NR	NR	1986(10)	Unclear	All-cause mortality	Young,28.0–30.8;Old,≥ 29.9	Young<25.5,25.5–27.9,28.0–30.8,≥30.9	Sex, age, diabetes duration, diabetes treatment, smoking, hypertension, and fasting plasma glucose	Measured	Young8.6
									Old<24.7,24.7–26.9,27.0–29.8,≥29.9			Old11.8
Eeg-Olofsson et al.(Sweden)	2009(13,087)	Prospective	60.3	44.3	1996(5.6, mean)	Yes	All-cause mortality	18–25	<25,25–29.9,≥30	Age, sex, type of hypoglycemic treatment, diabetes duration, smoking, HbA_1c_, systolic BP, antihypertensive drugs, lipid-lowering drugs, microalbuminuria	Measured	8.6
Khalangot et al.(Ukraine)	2009(89,443)	Prospective	62	66	1997(3)	No	All-cause mortality	25–30	<23,23–24.9,25–29.9,30–34.9,≥35	Age, smoking, alcohol consumption, systolic BP, total cholesterol, history of cardiovascular disease, diabetes treatments and duration of diabetes	Measured	<10,
												51.9–64.4^a^
Sluik et al.(Denmark, Germany, Italy, the Netherlands, Spain, Sweden)	2011(5435)	Prospective	57.3	46	1992(9.3, median)	No	All-cause and cardiovascular mortality	Men, ≥31.9	Male<24.9,25–27.1,27.2–29.1,29.2–31.8,≥31.9	Age, diabetes duration, insulin treatment, prevalent myocardial infarction, stroke, cancer, smoking status, smoking duration, smoking intensity, educational level, physical activity, alcohol consumption, quintiles of waist/height ratio or quintiles of BMI	Measured	3–5 (median values)
								Women, ≥33.6	Female<24.7,24.8–27.6,27.7–30.3,30.4–33.5,≥33.6			
Tseng et al.(Taiwan)	2013(89,056)	Prospective	Deceased, 66.3 (10.2)Survived, 58.2 (11.2)	54.0	1995(12)	No	All-cause mortality	25–29.9	<18.5,18.5–22.9,23.0–24.9,25.0–29.9,≥30	Age, sex, diabetes duration, insulin use, hypertension, smoking, living region	Self-reported	Deceased9.11Survived6.31
Logue et al.(United Kingdom)	2013 (106,640)	Retrospective	56	45	2001(5)	No	All-cause and cardiovascular mortality	25–30	20–24.9,25–29.9,30–34.9,35–39.9,40–44.9,45–49.9	Age at BMI determination, smoking status	Measured	<1
Yano et al.(Japan)	2013(3641)	Prospective	53.7	66.5	1992(10.2)	No	All-cause and cardiovascular mortality	21.1–25.0	14.2–21.1,21.1–25.0,≥25.0	Sex, current smoking status, systolic BP values, pre-existing myocardial infarction, stroke, or cancer	Measured	NR
Jackson et al.(United States)	2013(34,805)	Prospective	50.1	57	1997(9)	No	All-cause mortality	22.84–25.09	15.02–22.83,22.84–25.09,25.1–27.46,27.47–31.02,31.03–54.92	Age, marital status, smoking status, leisure-time physical activity, alcohol consumption, poor income, region of country, and self-reported general health status	Self-reported	8.7
Zhao et al.(United States)	2014(34,832)	Prospective	47.7–56.7,(Range of mean values)	47.6–79.2,(Range of mean values)	1997(8.7)	Yes	All-cause mortality	30–34.9	18.5–22.9,23–24.9,25–29.9,30–34.9,35–39.9,≥40	Age, sex, type of insurance, income, smoking, HbA_1c_, LDL cholesterol, systolic blood pressure, glomerular filtration rate, use of antihypertensive drugs, glucose-lowering agents, cholesterol-lowering agents	Measured	<1
Murphy et al.(Iceland)	2014(637)	Prospective	66–96	44.7	1967(6.7)	Yes	All-cause mortality	≥30	18.5–24.9,25–29.9, ≥30	Age, sex, education, duration of diabetes, midlife BMI, waist circumference, total cholesterol, HDL cholesterol, systolic BP, smoking status, hypertension, statin use, diabetes medication type, microalbuminuria, CRP	Measured	Normal weight10.0Overweight3.0Obese3.0(median)
Thomas et al.(United Kingdom)	2014(37,272)	Retrospective	60	47	1990(5, median)	Yes	All-cause mortality	≥30	<25,25–30,≥30	Age, sex, smoking status, systolic blood pressure, diastolic blood pressure, HbA_1c_, LDL, HDL, triglyceride measures	Measured	<1
Bozorgmanesh et al.(Iran)	2014(1322)	Prospective	53.7	55	1999(9.1)	No	All-cause mortality	26.9–31.1	15.7–26.9,26.9–31.1,31.1–57.7	Waist circumference, general CVD risk	Measured	<1
Tobias et al.(United States)	2014(11,427)	Prospective	61	73	1976(16)	Yes	All-cause and cardiovascular mortality	22.5–25.0	18.5–22.4,22.5–24.9,25.0–27.4,27.5–29.9,30.0–34.9,≥35.0	Age, race, marital status, menopausal status (for the NHS cohort only), presence or absence of a family history of diabetes, smoking status, alcohol intake, Alternate Healthy Eating Index score, physical activity	Self-reported	<1
Costanzo et al.(United Kingdom)	2015(10,568)	Prospective	63	46	1995(10.6)	No	All-cause mortality	25–29.9	<18.518.5–24.9,25–29.9, 30–34.9,≥35	Age, sex, duration of diabetes, systolic blood pressure, smoking, and comorbid conditions (such as cancer, chronic obstructive pulmonary disease, and chronic renal failure)	Measured	1

Abbreviations: BMI, body-mass index; BP, blood pressure; CRP, C-reactive protein; CVD, cardiovascular disease; HbA_1c_, glycated hemoglobin; HDL, high-density lipoprotein; LDL, low-density lipoprotein; NR, not reported; NHS, nurses’ health study

^a^ Diabetes duration was presented as the percentage of participants who had diabetes durations <10 years according to BMI categories and sex.

The findings of the included studies included different BMI categories. Only eight studies chose categories corresponding to the World Health Organization recommendation [3,16,18,19,21,22,24,26]. Among the three studies conducted in Asian countries, Tseng et al. [23] chose a BMI cutoff for overweight of >23 kg/m^2^, in line with recommendations for Asian populations [29]. A mean BMI of 47.5 kg/m^2^ was the highest assessed in the studies examining the association between BMI and all-cause mortality; thus, we could not evaluate results with a BMI exceeding 47.5 kg/m^2^. To account for the observed heterogeneity in exposure across the studies, generalized least squares for trend estimation was adopted to minimize the existing heterogeneity. This permitted prediction of the clinical outcomes of BMI across the whole range.

### Association between BMI and all-cause mortality

**Fig 2:**
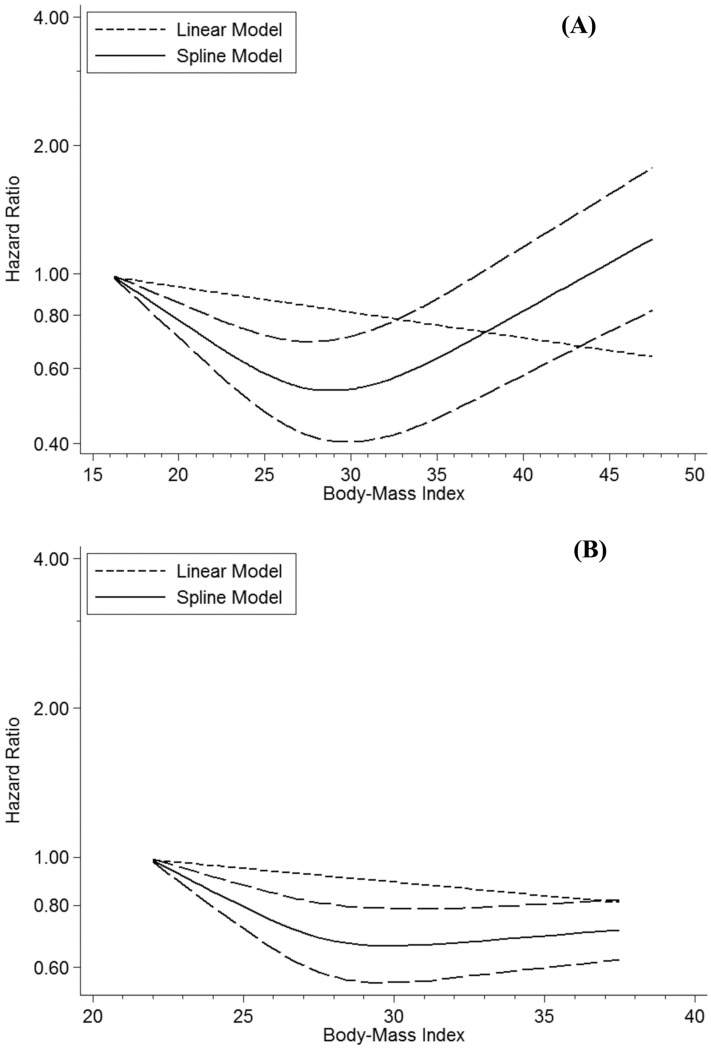
Dose-response associations between body mass index (BMI) and mortality among patients with type 2 diabetes. (A) Non-linear dose-response relationship between BMI and all-cause mortality (*P* < 0.001). (B) Non-linear dose-response relationship between BMI and cardiovascular mortality (*P* < 0.001). Non-linear and linear plots are displayed with continuous and medium-dashed black lines, respectively. Long-dashed black lines depict 95% confidence intervals. The log-scale of the hazard ratios are presented on the vertical axes.

When the random-effects dose-response analysis was performed for the 16 pooled studies, a significant non-linear relationship (*P* < 0.001) was observed between BMI and all-cause mortality among patients with type 2 diabetes with an estimated correlation matrix of −0.95 and estimated between-studies standard deviations (SDs) of 0.04 and 0.03. The risk of all-cause mortality among patients with type 2 diabetes decreased with an increase in BMI up to 28 kg/m^2^ but increased at BMIs exceeding 30 kg/m^2^ (Fig 2A). A two-stage random-effects model employed to compare the obtained results with the linear trend revealed that every 5 kg/m^2^ increase in BMI was significantly associated with a decreased risk of all-cause mortality (HR = 0.99, 95% CI = 0.97–1.00, *P* = 0.04). Furthermore, the large goodness-of-fit *P*-value (Q = 781.60, *P* < 0.0001) and the *I*^*2*^ value (*P* < 0.001, *I*^*2*^ = 97.1%) demonstrated between-study heterogeneity in the assumed linear relationship. No publication bias was present in the studies (Egger’s regression test *P*-value = 0.38).

### Association between BMI and cardiovascular mortality

In a pooled analysis of two studies providing data for 92,841 participants [18,25], a non-linear relationship was found between BMI and cardiovascular mortality in the random-effects dose-response analysis (*P* < 0.001; Fig 2B). The correlation matrix was calculated as −1, and the estimated between-studies SDs were 0.02 and 0.02. Our findings on the association between BMI and cardiovascular mortality revealed a non-linear, slightly increased risk at BMI > 31 kg/m^2^ and a decreased non-linear trend at BMI = 22–29 kg/m^2^ with a nadir at 29–31 kg/m^2^. To compare the observed results with the linear trend, a two-stage random-effects model was tested. The results suggested that a 5 kg/m^2^ increase in BMI was not significantly associated with a decreased risk of cardiovascular mortality (HR = 0.98, 95% CI = 0.85–0.13, *P* = 0.79). The results for goodness-of-fit (Q = 54.95, *P* < 0.0001) and the *I*^*2*^ statistic (*P* = 0.39, *I*^*2*^ = 1.4%) demonstrated that heterogeneity was not significant among the studies. Because of insufficient research on the relationship between BMI and cardiovascular mortality, proper subgroup and sensitivity analyses could not be performed.

### Possible sources of heterogeneity among studies

**Fig 3:**
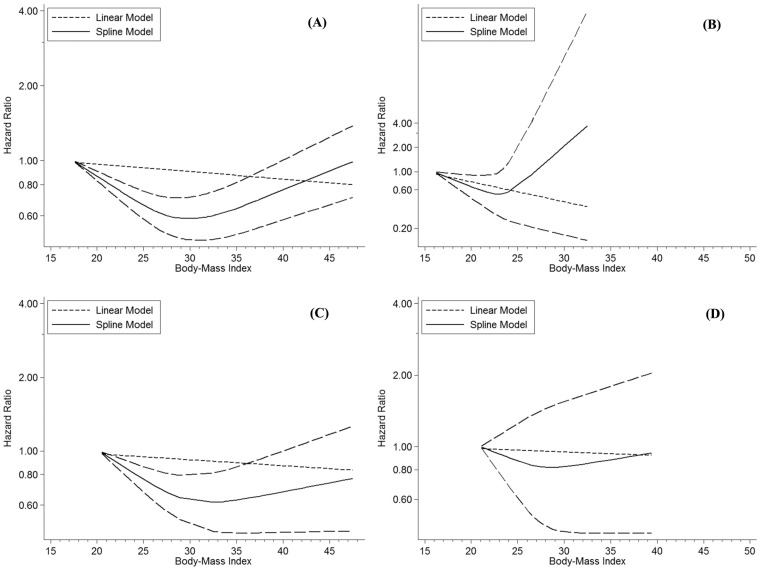
Subgroup analysis plots displaying non-linear dose-response relationships between body mass index (BMI) and all-cause mortality among patients with type 2 diabetes using pooled data from studies performed in (A) Western countries or (B) Asia, as well as those involving (C) patients with incident diabetes and (D) patients without smoking histories.

Subgroup analyses to detect the sources of heterogeneity determined that although heterogeneity was present, variations in the shapes of the slopes were not detected in any subgroup analysis (S1 Fig) excluding those for study location (Western or Asian), diabetes duration (incident diabetes: yes or no), and smoking history (yes or no). Variations in the shapes of the slopes were not evident in subgroup analyses of studies performed in Western countries (Fig 3A). In the subgroup of studies performed in East Asian countries, the HR displayed a U-shape increase with a BMI nadir of 22–23 kg/m^2^ (Fig 3B). When subgroup analysis was performed for studies that enrolled patients with incident diabetes [3,14,21,22,24], the HR at BMI > 34 kg/m^2^ increased, whereas at BMI < 32 kg/m^2^, the risk was reduced with a BMI nadir of 32–34 kg/m^2^ (Fig 3C). When subgroup analysis was conducted for studies involving patients without smoking histories [19,22,27], no significant non-linear relationship was found between BMI and all-cause mortality among patients with type 2 diabetes (Fig 3D). In the assumed linear relationship, a 5 kg/m^2^ increase in BMI was not associated with a reduction in all-cause mortality among patients with type 2 diabetes (HR = 1.00, 95% CI = 0.96–1.04). Considering that heterogeneity in a dose-response meta-analysis is linked to the shape of the relationship [12], our result was consistent with the main outcome in the sensitivity analysis.

## Discussion

In the current meta-analysis, a significant non-linear association was observed between BMI and all-cause mortality among patients with type 2 diabetes. With a BMI nadir of 28–30 kg/m^2^, the risk of all-cause mortality displayed a U-shape increase. With a BMI nadir of 29–31 kg/m^2^, the slope of cardiovascular mortality exhibited a gradual non-linear increase at BMI > 31 kg/m^2^. Additional subgroup analyses suggested that study location, diabetes duration, and smoking history might have contributed to heterogeneity among studies. Recently, another meta-analysis [30] assessed the association between BMI and mortality in patients with type 2 diabetes and concluded that a 5 kg/m^2^ increase in BMI was associated with a 5% reduction in the risk of all-cause mortality (HR = 0.95, 95% CI = 0.93–0.97). However, this study did not consider the possibility of a non-linear association between BMI and mortality. Moreover, the variables for which the researchers performed subgroup analyses were insufficient to illustrate the influence of important factors, i.e., diabetes duration and smoking history, on the total effect size. To our knowledge, our study is the first meta-analysis to document a non-linear association between BMI and mortality among patients with type 2 diabetes and conduct separate analyses of all-cause and cardiovascular mortality.

### Potential explanations and implications

The age-related explanations proposed for the obesity paradox could be potential reasons for the good prognosis of overweight and obese patients with diabetes aged 47–96 years in respective studies. A relevant issue is that body composition changes with age. While muscle and bone mass decrease with age, overall fat mass and fatty infiltration of muscles increase. These changes result in a lower overall BMI but not necessarily a metabolically favorable body composition. A study using dual X-ray absorptiometry identified higher fat mass and volumes of visceral fat and lower muscle mass in older adults [31]. This phenomenon, termed sarcopenic obesity, is a key factor associated with frailty, which could predispose older people to higher mortality [2]. Consequently, the prevalence of diabetes among normal-weight individuals increases as they age [2,5]. Consistent with this finding, two previous studies observed the obesity paradox phenomenon in older populations, but not younger ones [25,28].

Our findings imply that type 2 diabetes associated with metabolic stress related to obesity may differ from that associated with normal-weight [32]. Obese patients with type 2 diabetes might not have diabetes if they lose weight [33]. Those with higher genetic susceptibility to type 2 diabetes may be more prone to developing the disease at a lower metabolic stress of BMI, and they might also be at higher risk for complications or other diseases, which is associated with a poorer prognosis [2]. If this is true, then even if an obese patient with type 2 diabetes has a better prognosis than a normal-weight patient with diabetes, the prognosis might be further improved by losing weight. Asian patients with diabetes have been suggested to have lower BMI at the onset of diabetes and decreased β-cell function compared with European and American patients [34]. As β-cell function is more likely to be affected by genetic factors, ethnic differences appear to exist [35].

All but five of the studies [3,14,21,22,24] used prevalent type 2 diabetes to determine disease status. However, some patients successfully lost weight after a diagnosis of type 2 diabetes. These patients could not be considered normal-weight individuals with diabetes, and their mortality may have been influenced by their baseline and current weight simultaneously. Moreover, it is difficult to assess the duration of diabetes accurately. Patients with long-standing type 2 diabetes may lose weight following behavioral therapy, pharmacotherapy, or comorbidities, which could obscure the true association between normal-weight diabetes and mortality. Therefore, we performed subgroup analyses to compare mortality according to BMI at the time of incident adult-onset diabetes. When subgroup analysis was performed among incident diabetes studies, the slope of the HRs at BMI > 34 kg/m^2^ increased. At BMI < 32 kg/m^2^, the risk was reduced with a BMI nadir of 32–34 kg/m^2^ (Fig 3C). The altered shape of the relationship suggests that the heterogeneity among studies was included in the dose-response meta-analysis [12].

Smoking is a concern when analyzing weight status and mortality because of its association with decreased body weight and increased mortality [36]. Statistical adjustment for smoking status should be addressed comprehensively to control for varying levels of smoking duration and intensity, and this might induce other aspects of heterogeneity that reduce the validity of our findings. In the subgroup analysis of studies with participants who had never smoked, no significant linear or non-linear relationship was identified between BMI and all-cause mortality, whereas a non-linear relationship was observed in studies of patients with histories of smoking. Whether this effect modification suggests biological differences between smokers and nonsmokers or is due to methodological bias remains unclear [36].

Although type 2 diabetes among normal-weight individuals remains relatively uncommon, it is becoming more prevalent in population groups susceptible to normal-weight type 2 diabetes (i.e., older populations, non-white ethnic groups). Accordingly, management strategies could differ from the recommendations issued for overweight and obese people with type 2 diabetes. Currently, weight control through dietary modification and physical activity is recommended for overweight or obese patients with type 2 diabetes. Although physical activity may induce positive changes in blood sugar control, the goal in normal-weight patients may not be weight reduction, which could increase all-cause and cardiovascular mortality based on this meta-analysis. One study reported that cardiorespiratory fitness could be an even stronger predictor of mortality experience than weight status when the two variables are simultaneously included in the multivariable model [37]. Therefore, improving cardiorespiratory fitness through aerobic activities could be recommended for normal-weight patients with type 2 diabetes, but weight status should be maintained with increased calorie consumption accordingly. Attention is therefore shifting from weight to general fitness, as obesity may not be an exact surrogate for a participant’s level of fitness [38].

### Study strengths and limitations

There are several limitations to the current meta-analysis. The first concern is reverse causation, by which underlying chronic disease both causes weight loss and increases mortality. Referring to the issue of reverse causation, one study [39] concluded that the effect of potential bias was minimal based on their observation of few appreciable differences after either adjustment or exclusion of early deaths during the follow-up period. Similarly, findings from the subgroup analyses according to the exclusion of early death during the follow-up period in the present study did not change the main outcomes. Second, although most studies included in the analysis adjusted for potential covariates, there is a possibility that factors that were not measured might have been responsible for the observed association. Third, some studies used self-reported BMI, whereas others included standardized measurements [22,23]. Self-reported BMI has been strongly correlated with measured BMI; however, it has tended to be underestimated [40]. Despite the possibility for misclassification, we could not observe differential findings based on subgroup analysis according to the methods of BMI measurement. Fourth, as BMI data assessed at baseline might change during the follow-up period, it is not possible to determine the influences of changes in BMI on mortality. Finally, most of the studies could not address whether patients had type 2 diabetes or some other less common forms of diabetes in adults (e.g., type 1 diabetes, latent autoimmune diabetes). Moreover, overlap exists among even the most typical cases of diabetes. In addition, the current diabetes classification system presents challenges to the diagnosis and treatment of patients with diabetes, in part due to its conflicting and confounding definitions of type 1 diabetes, type 2 diabetes, and latent autoimmune diabetes of adults [41]. Despite these limitations, our study suggests that independent of diabetes type, normal-weight status among patients with diabetes may be a straightforward marker to predict increased mortality risk.

The strengths of the study include the assessment of the probability of non-linear relationships, the assessment of studies with varying BMI categories using generalized least squares for trend estimation, the exploration of cohort records exclusively to decrease the likelihood of heterogeneity, analysis of the effects of BMI on all-cause mortality and cardiovascular mortality among patients with type 2 diabetes, and the use of adjusted risks of death.

## Conclusions

Significant non-linear trends were observed in both of our dose-response meta-analyses, namely, in the 16 cohort studies assessing the effect of BMI on all-cause mortality among patients with type 2 diabetes and in the two cohort studies evaluating the influence of BMI on cardiovascular mortality. Study location, diabetes duration, and smoking history may have contributed to heterogeneity among studies that assessed obesity paradox phenomena among patients with type 2 diabetes according to subgroup analyses.

## Supporting Information

S1 Appendix Search strategy. (DOCX)

S2 Appendix PRISMA checklist. (DOC)

S1 Fig Subgroup analysis plots of a) males, b) females, c) prospective studies, d) subjects <65 years old, e) studies with more than 10,000 participants, f) studies with fewer than 10,000 participants, g) studies with more than 10 years of follow-up, and h) studies with less than 10 years of follow-up. (PDF)

S1 Table Newcastle-Ottawa Scale for bias risk in cohort studies. (DOCX)
