# Supplementary material for: Body Mass Index-Related Mortality in Patients with Type 2 Diabetes and Heterogeneity in Obesity Paradox Studies: A Dose-Response Meta-Analysis
Source: PLoS One. 2017 Jan 3;12(1):e0168247. doi: 10.1371/journal.pone.0168247 (PMC5207428; doi:10.1371/journal.pone.0168247)
Supplement: S1 Appendix — (DOCX) [file pone.0168247.s001.docx]

**Supplement 1.** Search strategy

**MEDLINE**

1. “obesity”[MH] OR “adiposity”[MH] OR “body mass index”[MH] OR “body size”[MH]

2. “obesity”[ALL] OR “obese” [ALL] OR “overweight” [ALL] OR “adiposity” [ALL] OR “adipose” [ALL] OR “adipos*” [ALL] OR “fat mass” [ALL] OR “body fat” [ALL] OR “body composition” [ALL] OR “body mass index” [ALL] OR “body size” [ALL] OR “body weight” [ALL] OR “BMI” [ALL] OR “body mass”[ALL]

3. 1 OR 2

4. “diabetes mellitus”[MH] OR “diabetes mellitus, type 2”[MH]

5. “diabetes mellitus”[ALL] OR “diabetes”[ALL] OR “type 2 diabetes”[ALL]

6. 4 OR 5

7. 3 AND 6

8. “mortality”[MH] OR “death”[MH]

9. “mortality”[ALL] OR “mortalities”[ALL] OR “death*”[ALL] OR “survive”[ALL] OR “surviv*”[ALL] OR “prognosis”[ALL] OR “progno*”[ALL] OR “paradox”[ALL] OR “all-cause”[ALL] OR “cardiovascular disease”[ALL] OR “cardiovascular”[ALL] OR “coronary heart disease”[ALL] OR “coronary”[ALL] OR “stroke”[ALL] OR “cerebrovascular disease”[ALL]

10. 8 OR 9

11. 7 AND 10

12. “humans”[MH]

13. 11 AND 12

MH = MeSH terms, ALL = All fields

**Limitation:** humans

**Date of Search:** Dec. 5 2015

**Results:** 33156 articles were found

**EMBASE**

1. ‘obesity’/exp OR ‘obesity’/syn

2. ‘body mass’/syn OR ‘fat mass’/syn OR ‘body fat’/syn OR ‘body composition’/syn OR ‘body size’/syn OR ‘body weight’/syn

3. 1 OR 2

4. ‘diabetes mellitus’/exp OR ‘diabetes mellitus’/syn

5. ‘non insulin dependent diabetes mellitus’/syn

6. 4 OR 5

7. 3 AND 6

8. ‘mortality’/syn OR ‘death’/syn OR ‘survival’/syn OR ‘prognosis’/syn OR ‘obesity paradox’ OR ‘cardiovascular disease’/syn OR ‘ischemic heart disease’/syn OR ‘cerebrovascular accident’/syn OR ‘cerebrovascular disease’/syn

9. ‘longitudinal study’/syn OR ‘retrospective study’/syn OR ‘prosepctive study’/syn OR ‘observational study’/syn OR ‘follow up’/syn OR ‘cohort analysis’/syn

10. [humans]/lim AND [embase]/lim

11. 7 AND 8 AND 9 AND 10

Exp = explosion search, syn = synonymous search, lim = limitation

**Limitation:** humans

**Date of Search:** Dec. 5 2015

**Results:** 22160 articles were found

**CENTRAL database (The Cochrane Library)**

1. MeSH descriptor **Obesity** explode all trees

2. MeSH descriptor **Body Mass Index** explode all trees

3. (overweight) OR (fat mass) OR (body fat) OR (body composition) OR (body size) OR (body weight)

4. #1 OR #2 OR #3

5. MeSH descriptor **Diabetes Mellitus** explode all trees

6. MeSH descriptor **Diabetes Mellitus, Type 2** explode all trees

7. (diabetes mellitus) OR (diabetes) OR (type 2 diabetes) OR (fasting plasma glucose) OR (fasting blood sugar) OR (glycated hemoglobin)

8. #5 OR #6 OR #7

9. #4 AND #8

10. MeSH descriptor **Mortality** explode all trees

11. MeSH descriptor **Death** explode all trees

12. MeSH descriptor **Survival** explode all trees

13. (mortality) OR (death) OR (survival) OR (prognosis) OR (obesity paradox) OR (cardiovascular diseases) OR (coronary disease) OR (stroke) OR (cerebrovascular disease)

14. #10 OR #11 OR #12 OR #13

15. #9 AND #14

All field text searched

**Limitation:** none

**Date of Search:** Nov. 5 2015

**Results:** 2908 articles were found

A total 38342 articles were screened
