# Supplementary material for: Body Mass Index-Related Mortality in Patients with Type 2 Diabetes and Heterogeneity in Obesity Paradox Studies: A Dose-Response Meta-Analysis
Source: PLoS One. 2017 Jan 3;12(1):e0168247. doi: 10.1371/journal.pone.0168247 (PMC5207428; doi:10.1371/journal.pone.0168247)
Supplement: S1 Fig — (PDF) [file pone.0168247.s003.pdf]

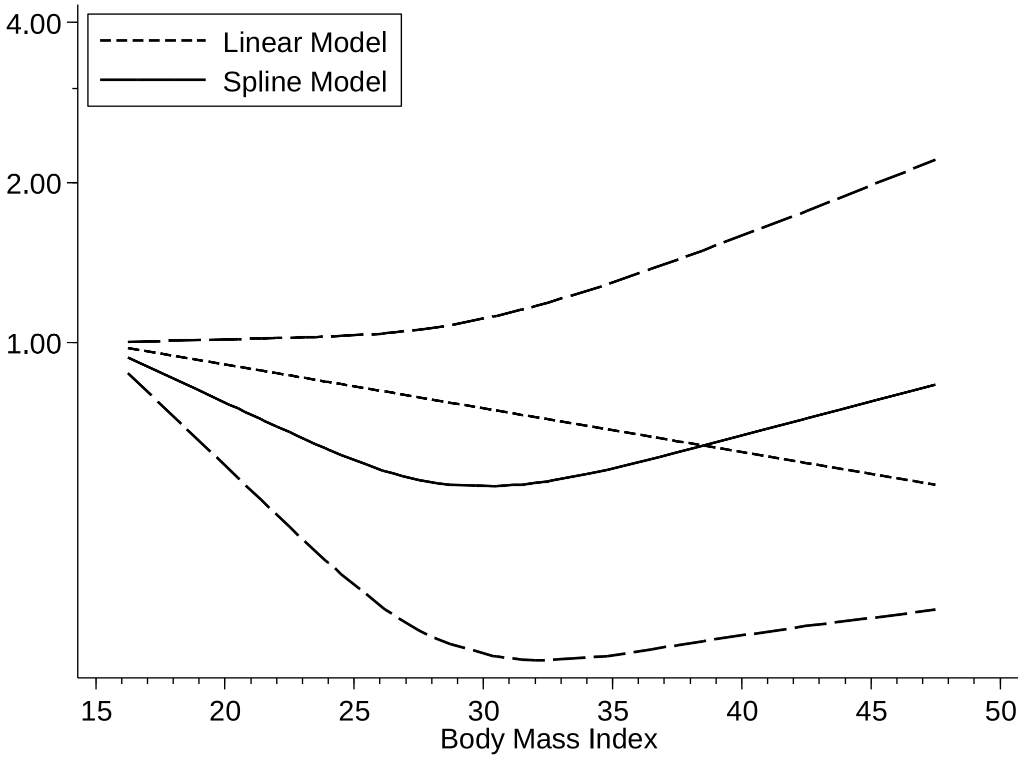

Supplement Figure 1A. Subgroup analysis plot (Male)

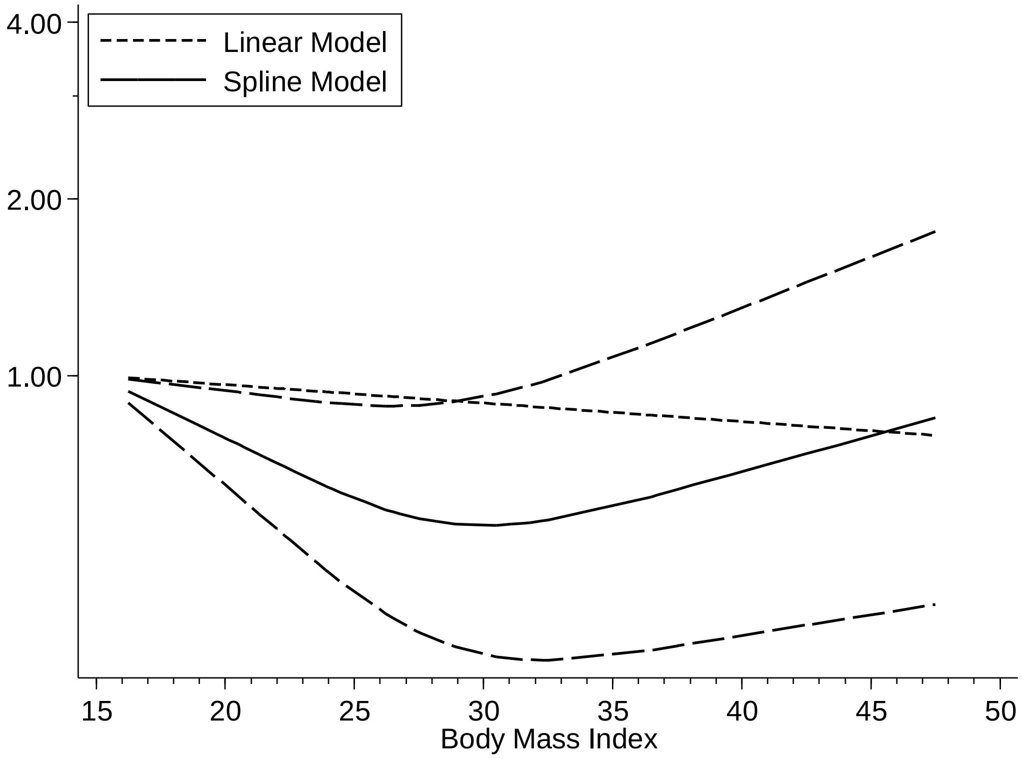

Supplement Figure 1B. Subgroup analysis plot (Female)

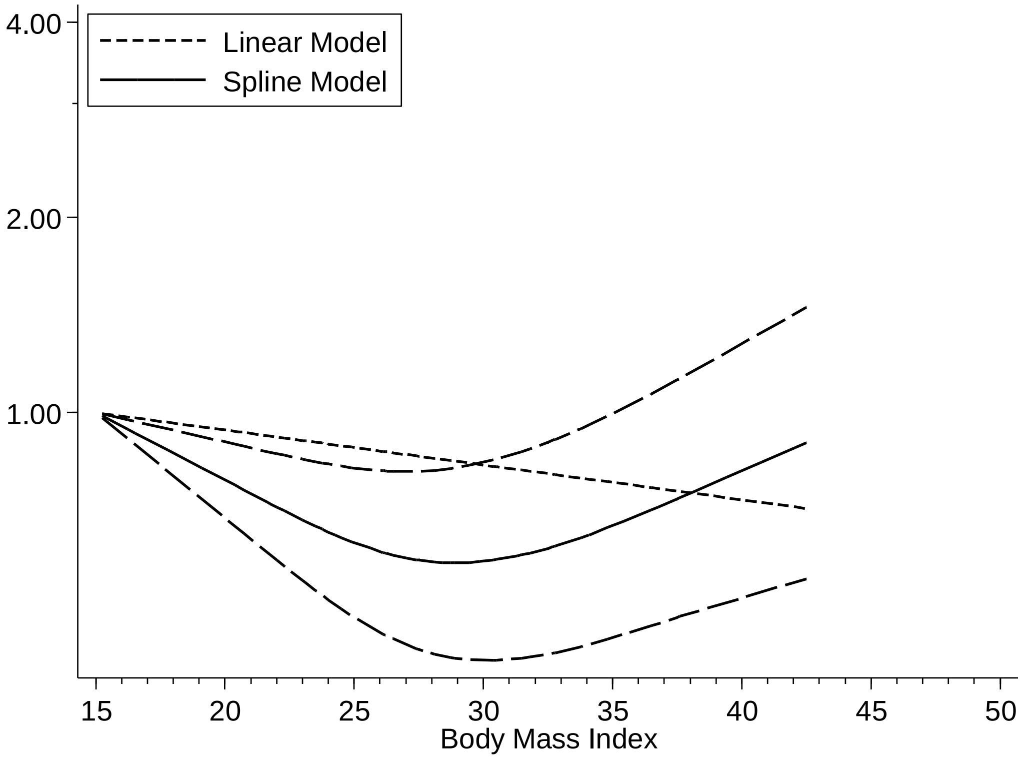

Supplement Figure 1C. Subgroup analysis plot (Prospective studies)

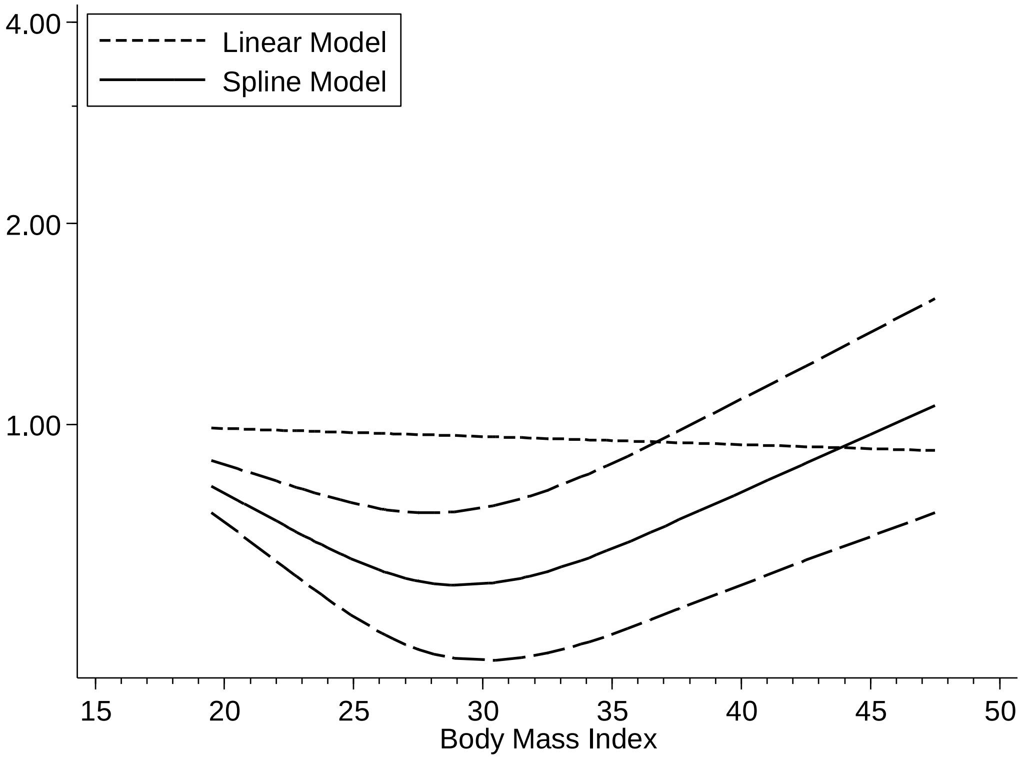

Supplement Figure 1D. Subgroup analysis plot (less than 65 years old)

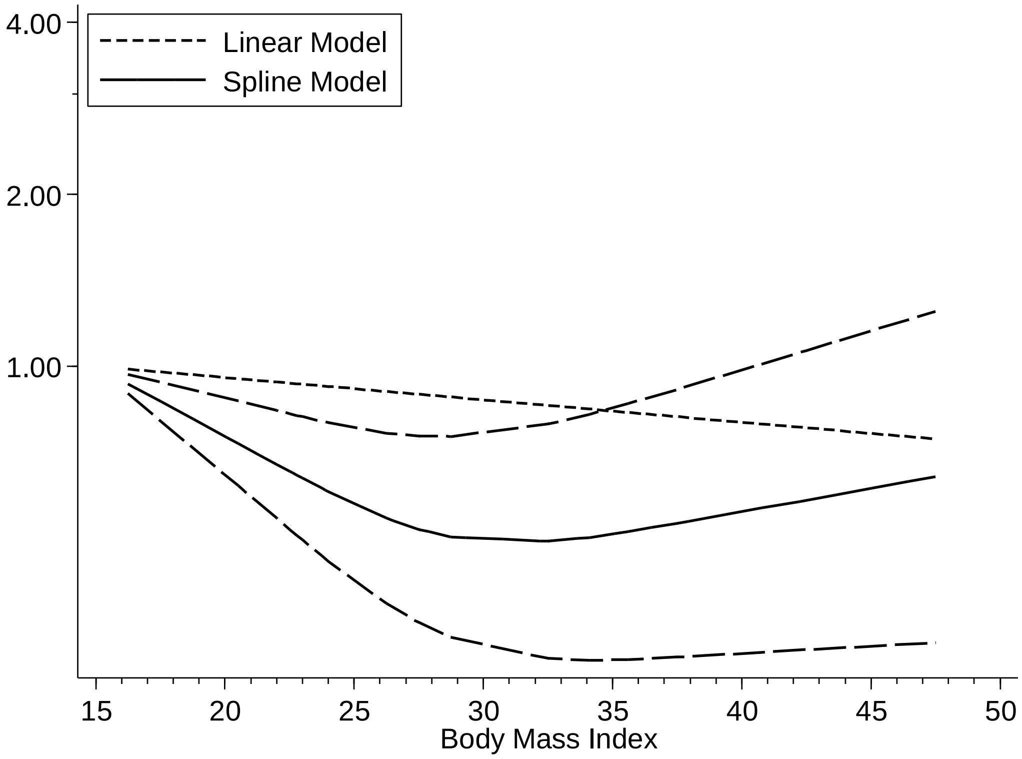

Supplement Figure 1E. Subgroup analysis plot (Studies that included more than 10,000 participants)

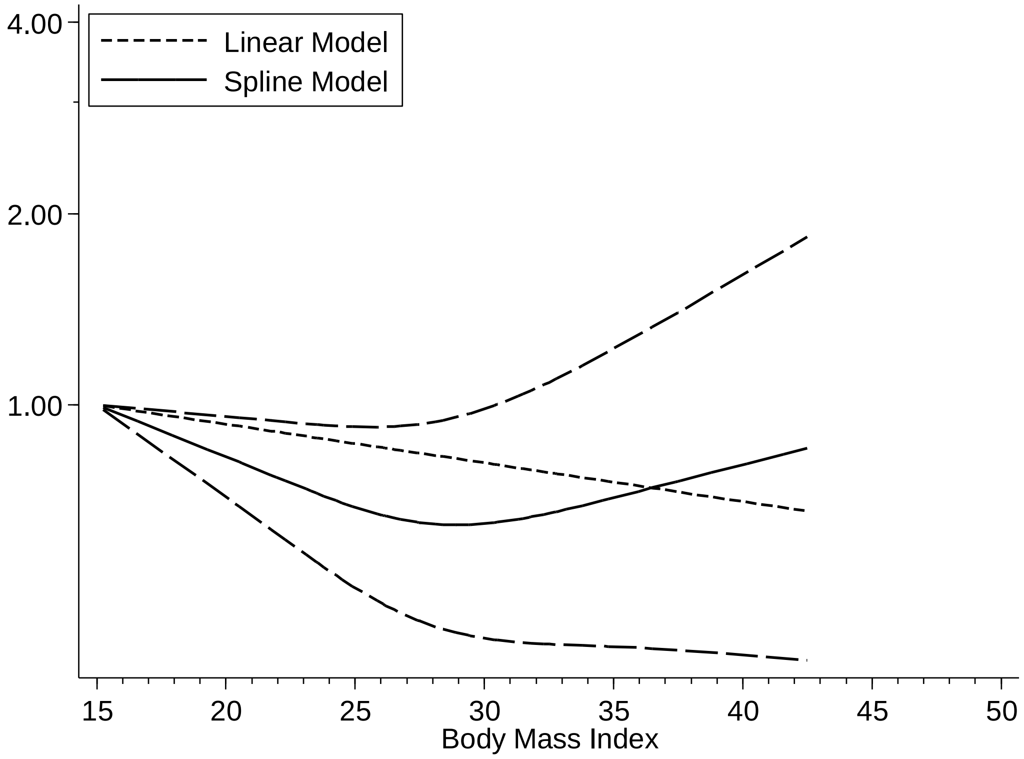

Supplement Figure 1F. Subgroup analysis plot (Studies that included less than 10,000 participants)

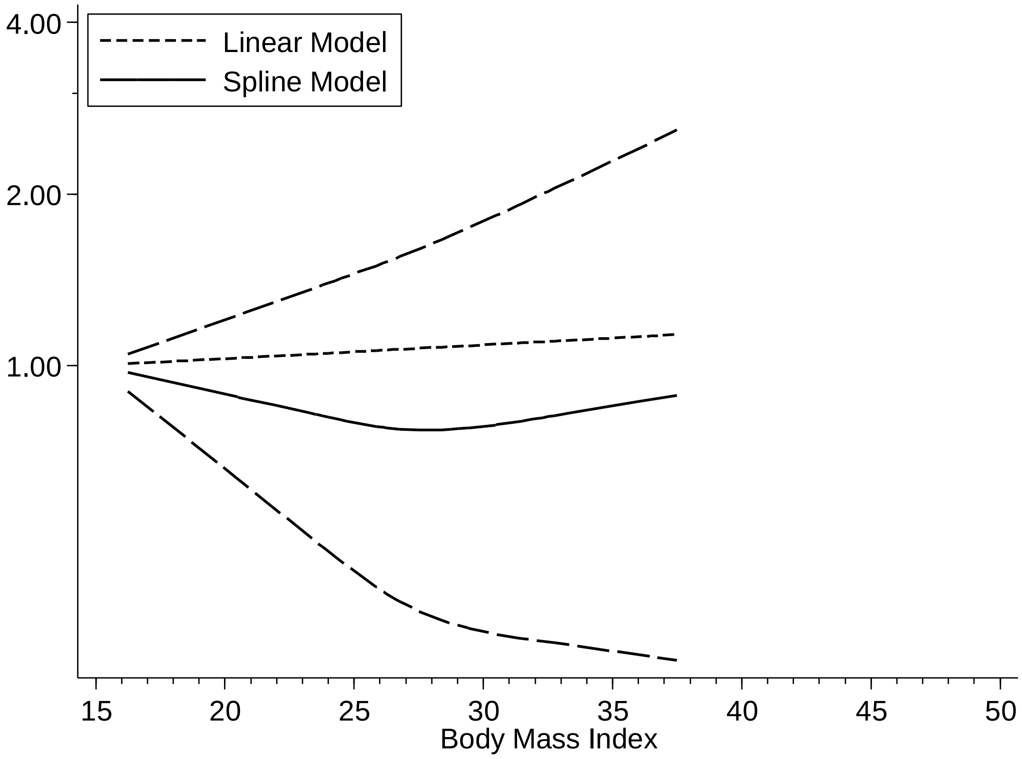

Supplement Figure 1G. Subgroup analysis plot (follow-up period, longer than 10 years)

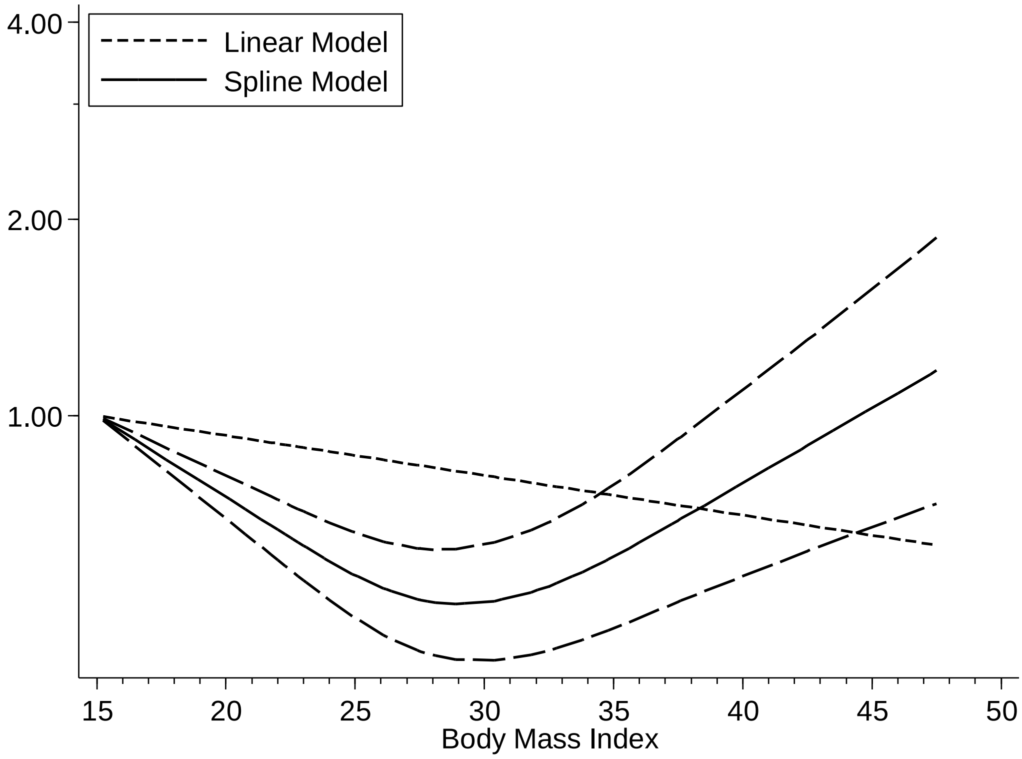

Supplement Figure 1H. Subgroup analysis plot (follow-up period, shorter than 10 years)
