# Supplementary material for: Body Mass Index-Related Mortality in Patients with Type 2 Diabetes and Heterogeneity in Obesity Paradox Studies: A Dose-Response Meta-Analysis
Source: PLoS One. 2017 Jan 3;12(1):e0168247. doi: 10.1371/journal.pone.0168247 (PMC5207428; doi:10.1371/journal.pone.0168247)
Supplement: S1 Table — (DOCX) [file pone.0168247.s004.docx]

| **Supplement Table 1. Newcastle-Ottawa Scale for bias risk in cohort studies** | | | | | | | |
| --- | --- | --- | --- | --- | --- | --- | --- |
|  | **Adequacy of selection** | | | **Comparability** | **Outcome assessment** | | |
|  | **Representativeness**  **of the exposed cohort** | **Selection of**  **the non-exposed cohort** | **Ascertainment**  **of exposure** |  | **Assessment**  **of outcome** | **Follow-up period**  **was sufficiently long**  **for outcome to occur**  **(>5 years)** | **Adequacy of**  **follow-up period**  **among cohorts**  **(>80%)** |
| Ford et al. 1991 | * | * | * | ** | * | * | * |
| Chaturvedi et al. 1995 | * | * | * | ** | * | * | * |
| Zoppini et al. 2003 | * | * | * | ** | * | * | * |
| Eeg-Olofsson et al. 2009 | * | * | * | ** | * | * | * |
| Khalangot et al. 2009 | * | * | * | ** | * |  | * |
| Sluik et al. 2011 | * | * | * | ** | * | * | * |
| Tseng et al. 2013 | * | * | * | ** | * | * | * |
| Logue et al. 2013 | * | * | * | ** | * | * | * |
| Yano et al. 2013 | * | * | * | ** | * | * | * |
| Jackson et al. 2013 | * | * | * | ** | * | * | * |
| Zhao et al. 2014 | * | * | * | ** | * | * | * |
| Murphy et al. 2014 | * | * | * | ** | * | * | * |
| Thomas et al. 2014 | * | * | * | ** | * | * | * |
| Bozorgmanesh et al. 2014 | * | * | * | * | * | * | * |
| Tobias et al. 2014 | * | * | * | ** | * | * | * |
| Costanzo et al. 2015 | * | * | * | ** | * | * | * |
| Asterisks represent the star ratings as per the Newcastle-Ottawa Scale.  * and ** indicate the highest ratings for these categories. | | | | | | | |
